# Clinical and Radiographic Outcomes of Modified Multi-Tooth Posterior Maxillary Alveolar Segment Infracture Without a Lateral Window for Staged Implant Placement: A Prospective Case Series

**DOI:** 10.3390/jcm15197516

**Published:** 2026-09-27

**Authors:** Roman Šmucler, Simona Dianišková, Justina-Anna Šmuclerová

**Affiliations:** 1 Asklepion-Lasercentrum, Londýnská 39, 120 00 Prague, Czech Republic; 2 Department of Stomatology, Faculty of Medicine, Slovak Medical University, 833 03 Bratislava, Slovakia; 3 Third Faculty of Medicine, Charles University, 100 00 Prague, Czech Republic; justina.smucler@gmail.com

**Keywords:** posterior maxilla, maxillary sinus, alveolar segment infracture, sinus floor elevation, tooth extraction, vertical augmentation, dental implants, cone-beam computed tomography

## Abstract

**Background/Objectives:** This prospective case series evaluated whether modified multi-tooth posterior maxillary alveolar segment infracture without a lateral window permitted staged implant placement. **Methods:** Of 53 patients assessed, 8 were excluded and 25 declined; 20 eligible consenting non-smokers were enrolled consecutively and treated at 26 sites. Following three adjacent extractions, a single bone block containing the extraction sockets was mobilized through an oral-side oval osteotomy, displaced superiorly, and augmented. Cone-beam computed tomography was obtained before augmentation, at 6 months, and 1 year after loading. **Results:** All 26 sites received two implants at 6 months; all 52 implants survived through 1 year of loading. Mean estimated displacement was 7.2 mm. Mean implant-bearing mineralized-tissue heights were 12.9 mm at 6 months and 12.8 mm at loaded follow-up; individual paired measurements and dispersion were unavailable. Pain lasted 4.7 ± 2.3 days. Small-to-moderate hematoma occurred in 10/20 patients. No dehiscence or clinically suspected membrane perforation was reported at 26 sites; no clinically suspected sinusitis or reoperation was reported in 20 patients. **Conclusions:** The findings support feasibility in selected patients, not comparative effectiveness, confirmed membrane integrity, or individual dimensional stability. Controlled studies with reproducible imaging and longer follow-up are required.

## 1. Introduction

Posterior maxillary rehabilitation may be complicated by post-extraction remodeling, reduced bone density, and sinus pneumatization. Treatment options include sinus floor elevation, ridge augmentation, short implants, or combinations of these approaches. Selection of a transcrestal or lateral-window approach depends on the available bone, sinus anatomy, required elevation, and anticipated morbidity [1,2,3,4].

A systematic review of sites with residual bone height ≤6 mm found no statistically significant differences between transcrestal and lateral approaches in implant survival, membrane perforation, or marginal bone loss; however, the included evidence had important risks of bias [2]. These findings do not establish equivalence for every anatomical indication. Avoiding a lateral window also limits direct visualization of the Schneiderian membrane, making the method of perforation assessment important when interpreting safety outcomes [3,4].

Superior displacement of local alveolar bone during tooth extraction is an established concept. Jensen et al. described intrusion of interradicular bone at maxillary molar extraction sites [5], and Block subsequently reported a socket-based modification [6]. Related approaches include two-step osteotome-mediated transalveolar sinus elevation and lateral displacement of the palatal wall combined with alveolar ridge splitting, compared in a three-year retrospective study by Crespi et al. [7]. Together, these reports provide the context for incremental modifications rather than a claim to a new biological principle.

Segmental inlay or sandwich osteotomy offers a further conceptual comparison because local bone is repositioned and graft material is placed in an interpositional space. Reviews describe potential vertical augmentation but also heterogeneity, complications, and limitations of the supporting evidence [8,9,10,11,12]. Anterior maxillary studies [13,14,15] and posterior maxillary reports [16,17,18] differ in defect anatomy, movement of the segment, grafting, and implant timing; their radiographic gains are not directly interchangeable with the measurements in the present series.

The present modification mobilizes a single contiguous segment, including the extraction sockets of three adjacent teeth, through an oral-side oval osteotomy without a lateral window. The primary aim was to assess whether this approach permitted placement of two planned implants per treated site. Radiographic findings, postoperative morbidity, and implant survival through 1 year of loading were also described.

## 2. Materials and Methods

### 2.1. Study Design, Ethics, and Registration

This prospective, single-center, single-arm interventional case series was conducted at Asklepion-Lasercentrum, Prague, Czech Republic. The study protocol was approved by the Asklepion Ethics Committee on 15 February 2023 (approval no. 2023/2), before recruitment began. Recruitment ran from 14 March to 21 November 2023, and treatment followed the approved protocol. All participants provided written informed consent for participation, treatment, and use of anonymized clinical and radiographic data. Reporting was reviewed against the PROCESS 2023 domains [19]; the accompanying reporting map (Supplementary Material S1) identifies both reported items and reporting limitations.

The study was not registered in a public trial registry. At study initiation, the investigators understood public registration to be required principally for externally funded research and did not arrange registration for this internally funded project. This explanation describes the investigators’ understanding; it is not presented as a formal exemption from prospective registration. Because treatment was assigned by the study protocol, the investigation is reported as prospective and interventional, not as a retrospectively reclassified observational study. Ethics approval and the absence of external grant funding do not substitute for public registration [20]. The original outcome hierarchy and analysis plan therefore cannot be independently checked against a prospectively registered protocol.

### 2.2. Participants and Eligibility Criteria

Twenty eligible, consenting non-smoking patients were enrolled consecutively, 12 men and 8 women, with a mean age of 62.3 ± 7.5 years (range, 47–79 years). Fourteen patients received unilateral treatment and six received bilateral treatment. A treated site is defined here as one posterior maxillary side containing the three adjacent extraction sites; the cohort therefore comprised 26 treated sites in 20 patients. Site counts are not used to imply 26 independent patients or 26 separate operative sessions.

Inclusion criteria were: (1) indication for simultaneous extraction of the maxillary second premolar, first molar, and second molar at the treated side (FDI positions 15–17 or 25–27); (2) close radiographic relationship of the posterior roots and alveolar segment to the maxillary sinus; (3) planned staged implant-supported fixed rehabilitation; (4) a preoperative vertical height of at least 7 mm at the intended implant-bearing point of the segment, permitting a planned superior displacement of at least 5 mm; and (5) willingness and ability to attend the full follow-up protocol.

Exclusion criteria were general or local contraindications to implant therapy, congenital or acquired coagulation disorders, medication-related impairment of hemostasis, diabetes mellitus, and other clinically relevant systemic diseases considered likely to impair surgical healing or osseointegration. Current or previous sinus disease was also an exclusion criterion, including previous sinusitis or sinus treatment even when considered successful. A history of oroantral communication was an additional exclusion criterion. An otorhinolaryngologist performed the assessment and determined exclusion on these grounds. The purpose was to reduce uncertainty in attributing subsequent sinus-related findings to the intervention. A standardized symptom inventory, quantitative threshold for radiographic mucosal changes, and individual baseline sinus findings were not available for this report.

Fifty-three potential participants were assessed for eligibility during the recruitment period. Eight were excluded by the otorhinolaryngologist because of previous or current sinusitis and/or a history of oroantral communication. The combined number of these exclusions is reported; separate counts for the individual conditions were not available. Of the remaining 45 patients, 25 declined the investigational operation because they considered it more invasive than conventional treatment and did not wish to undergo a research procedure. All 20 eligible patients who consented were enrolled consecutively. Refusal occurred before enrollment and is distinct from loss to follow-up.

### 2.3. CBCT Acquisition, Alignment, and Measurements

CBCT examinations were obtained before augmentation, 6 months after augmentation, and 1 year after functional loading using the same i-CAT Next Generation unit (Imaging Sciences International, Hatfield, PA, USA). Acquisition parameters reported by the treating center were a field of view of 16 × 16 cm, a nominal voxel size of 0.125 mm, a fixed tube voltage of 120 kVp, a tube current of 5 mA, and an exposure time of 3.7 s. The viewing software was CAT vision, version 3.0. These acquisition and software details were confirmed by the treating center during revision; scan-level DICOM metadata were not available for independent verification in this revision.

Serial volumes were compared using software-assisted alignment and side-by-side multiplanar review. Justina-Anna Šmuclerová (J.-A.Š.), who did not perform the operations, evaluated the radiographs and manually checked correspondence of the selected measurement location across examinations. A reproducible transformation model, alignment sequence, orientation convention, and fixed anatomical landmark set were not available in the measurement documentation. Blinding to examination time point and to clinical outcomes was not documented; the reader is therefore not described as blinded.

The measurements targeted the highest residual osseous point intended to support an implant rather than an average across the augmented region. In the original measurement description, the preoperative target was the apical osseous apex of the planned extraction alveolus. Height was measured along the intended implant trajectory from a crestal reference to the most superior continuous radiopaque/mineralized point. Lower peripheral portions of the contour were not included because they were not intended implant-bearing regions. This describes the original selected-point assessment; it does not establish a validated, independently reproducible anatomical measurement protocol.

Segment displacement was estimated by comparing the identified segment in the preoperative and 6-month examinations. It was therefore a radiographic estimate, not a direct measurement of immediate intraoperative displacement. Preoperative segment height, estimated displacement, and postoperative implant-bearing mineralized-tissue height describe different aspects of the reconstruction and are reported separately. CBCT radiopacity cannot determine the proportion of vital bone and residual graft; no histological samples were obtained.

Individual paired radiographic values, their dispersion, and the exact aggregation across the two intended implant positions per treated site are not available in the dataset used for this report. Consequently, the original number of observations contributing to each radiographic mean cannot be specified beyond their origin in the 26-site cohort. No repeated-measurement or intra-/interobserver reliability analysis was performed. The reported radiographic means are descriptive and do not establish measurement precision, individual dimensional stability, net bone gain, or volumetric preservation.

### 2.4. Surgical Technique

All augmentation operations were performed by Roman Šmucler. Antibiotic prophylaxis was administered 2 h before surgery and comprised amoxicillin 1750 mg plus clavulanic acid 250 mg (Augmentin; combined dose, 2000 mg). The formulation and route were not specified in the documentation available for this report. Local infiltration anesthesia used 4% articaine, with a volume of 4–6 mL. A buccal soft-tissue flap provided access and was lengthened and advanced using the Wassmund technique to permit closure.

The second premolar and first and second molars were removed as atraumatically as possible. Multi-rooted teeth were sectioned before removal to limit uncontrolled fracture of the surrounding alveolus. A rotary surgical bur (C254A.316.012; supplied by Hu-Fa Dental a.s., Otrokovice, Czech Republic) was then used from the oral/crestal aspect to create one oval circumferential osteotomy around the former tooth-bearing region. The outlined segment included the three extraction-socket complexes and their surrounding and intervening bone. It was not divided into three separate blocks.

The oval osteotomy was accessed only from the mouth. No lateral sinus window, separate lateral-wall osteotomy, or additional superior horizontal cut was made. The sinus-facing interface of the block was not surgically dissected free. Progressive infracture and superior displacement were achieved by controlled mallet taps transmitted through an osteotome from the oral side, with repeated reassessment of mobility and position. The entire block, including all three extraction-socket complexes, moved superiorly as one unit into the sinus region; the sockets did not remain behind in the original alveolar position. Advancement continued to the planned position with clinical reassessment of stability. No plates, screws, or other rigid fixation elements were used; retention relied on the surrounding soft tissues. Segment perfusion and a specific vascular pedicle were not measured.

The extraction sockets remained open toward the oral side after elevation. Their lumina and the newly created space beneath the displaced block were filled with SinossGraft, a particulate synthetic calcium-phosphate bone substitute (novadento Dental Products, Bad Saulgau, Germany), together with GENTA-COLL^®^ resorb, a gentamicin-containing resorbable collagen sponge (RESORBA Medical GmbH, Nürnberg, Germany). The augmented oral aspect was then covered with a separate SinossMem resorbable collagen membrane (novadento Dental Products, Bad Saulgau, Germany). No collagen membrane was interposed between the elevated block and the maxillary sinus. The soft tissues were closed using a continuous absorbable GLYCOLON suture (RESORBA Medical GmbH, Nürnberg, Germany). Figure 1 illustrates the conceptual sequence; implant placement was staged rather than simultaneous.

The Schneiderian membrane was not directly exposed or systematically visualized during the operation. Assessment for an oroantral communication included Valsalva testing and visual/instrumental inspection of the accessible operative wound. These clinical checks did not provide direct confirmation of membrane integrity. Numerical osteotomy depth, a quantitative segment-stability criterion, and standardized rescue criteria for an unstable segment or suspected membrane injury were not available for reporting.

### 2.5. Postoperative Care and Adverse-Event Assessment

Patients were instructed to apply chlorhexidine-digluconate gel locally three times daily for 3 weeks. Systemic antibiotics were not routinely continued after surgery but could be prescribed if clinically indicated. Patients were contacted by telephone every postoperative day while pain persisted. Pain duration was recorded as the number of days with patient-reported postoperative pain. Swelling was evaluated clinically but not measured with a validated scale. Hematoma was documented descriptively from routine clinical records as small-to-moderate or large; no formal severity index was used.

Clinical care included an otorhinolaryngological review after primary wound healing. Sinoscopy was reserved for clinical indications rather than mandated in every patient; the number of patients actually undergoing sinoscopy and a standardized indication checklist were not available for this report. The exact timing of the early clinical review was not specified. Later study stages comprised the 6-month pre-implant assessment, prosthetic rehabilitation after implant healing, and examination 1 year after loading.

Adverse events reported in this series were wound dehiscence, hematoma, clinically suspected sinusitis, reoperation, and clinically suspected Schneiderian membrane perforation. Perforation ascertainment combined the intraoperative clinical checks described above with the postoperative course. Sinusitis reporting refers to clinical suspicion during surgical follow-up and otorhinolaryngological assessment, not to systematic endoscopic exclusion of asymptomatic disease. A standardized symptom/sign definition and diagnostic score were not available. The absence of a clinically suspected complication cannot exclude occult membrane injury or subclinical sinus abnormalities.

### 2.6. Implant Placement and Prosthetic Rehabilitation

At 6 months, implant placement was undertaken after radiographic assessment of the intended implant-bearing region. The protocol reported a minimum CBCT height of 8 mm for proceeding. Two DENTIS OneQ Bone Level implants (DENTIS Co., Ltd., Daegu, Republic of Korea) were placed per treated site. Implant diameters were 4.5 or 5.2 mm and lengths were 10 or 12 mm, selected according to local anatomy. Placement was freehand rather than guided, with the insertion geometry individualized to each site. Quantitative implant-specific insertion-depth, angulation, and protrusion measurements were not available for reporting.

The 8 mm threshold was a criterion for proceeding, not proof that every 10 or 12 mm implant was completely surrounded by augmented mineralized tissue. Implant length and completed insertion were not used to calculate augmentation height or to validate the radiographic measurements. Although placement was individualized and freehand, the quantitative relationship between each implant’s length, position, and available osseous height cannot be reconstructed from the aggregate data.

All implants were managed with a one-stage transmucosal healing protocol using healing abutments for 3 months. Definitive fixed prosthetic reconstructions were then delivered as independent or splinted bridge units according to the prosthetic plan. Loading was scheduled 3 months after implant insertion; operational factors produced a maximum deviation of 30 days from the planned date.

### 2.7. Outcomes and Reporting Units

The main descriptive outcomes were implant-bearing mineralized-tissue height at 6 months, the proportion of treated sites receiving the two planned implants, and implant survival 1 year after loading. Survival was defined as the implant remaining in situ at that examination. It was not a validated composite endpoint incorporating peri-implant health, mobility, probing, suppuration, radiographic bone loss, and patient-reported function.

Additional outcomes were estimated segment displacement, mineralized-tissue height 1 year after loading, pain duration, wound dehiscence, hematoma, clinically suspected sinusitis, reoperation, clinically suspected membrane perforation, follow-up completion, and exploratory marginal crestal remodeling. Pain, hematoma, sinusitis, reoperation, and follow-up completion were reported per patient; dehiscence, suspected perforation, and staged-placement feasibility were reported per treated site; implant retention and dimensions were reported per implant. Radiographic summaries concern the selected implant-bearing location, not whole-region volume.

### 2.8. Statistical Analysis

No a priori sample-size calculation was performed. Analyses were descriptive. Continuous variables are reported as mean ± standard deviation where available; otherwise, only the recorded mean or exploratory range is presented. When individual observations were unavailable, dispersion and paired changes could not be calculated. No hypothesis test, equivalence test, or cluster-adjusted longitudinal model was fitted to the radiographic summaries because the necessary individual paired observations were unavailable.

The 26 sites were nested within 20 patients, and the 52 implants were nested within sites and patients. Site-level and implant-level percentages are descriptive, without confidence intervals that assume these observations are independent. As a post hoc patient-level summary added during revision, the proportion of patients retaining all their implants was calculated with an exact two-sided 95% Clopper–Pearson confidence interval, using the 20 patients as the independent observational units. This interval estimates patient-level complete implant retention, not individual-implant survival and not an adjusted implant-level effect.

## 3. Results

### 3.1. Cohort and Follow-Up

Of 53 patients assessed, 8 were excluded on otorhinolaryngological grounds and 25 declined the investigational operation; 20 eligible consenting patients were enrolled consecutively. All 20 completed augmentation, implant placement, prosthetic rehabilitation, and the 1-year post-loading examination. No enrolled patient was lost to follow-up. The 14 unilateral and six bilateral cases yielded 26 treated sites. Follow-up is reported relative to functional loading; the calendar date of the last examination was not available in the report dataset. Recruitment flow and baseline characteristics are presented in Table 1.

### 3.2. Early Clinical Outcomes and Staged-Placement Feasibility

Small-to-moderate hematoma was documented in 10/20 patients (50%); no large hematoma was reported. Clinically pronounced swelling was commonly observed but not quantified. Mean patient-reported pain duration was 4.7 ± 2.3 days. No patient required postoperative systemic antibiotics, reoperation, or treatment for clinically suspected maxillary sinusitis. No dehiscence or clinically suspected membrane perforation was reported at the 26 treated sites. These clinical observations do not confirm membrane integrity. Event counts and denominators are presented in Table 2.

All 26 sites received the two planned implants at the 6-month stage, giving a descriptive site-level feasibility proportion of 26/26 (100%). The reported radiographic threshold for proceeding was met at each site. This endpoint records that planned implant placement occurred; it is not independent validation of augmentation height, full intrabony implant accommodation, or superiority over another technique.

### 3.3. Radiographic and Implant Outcomes

Mean preoperative segment height was 9.4 mm and mean estimated superior displacement was 7.2 mm. Mean implant-bearing mineralized-tissue height was 12.9 mm at 6 months and 12.8 mm at 1 year after loading (Table 3). The arithmetic difference between the two rounded follow-up means was 0.1 mm; this is not a paired estimate of individual change. Dispersion and individual paired values were unavailable for these radiographic summaries.

Exploratory marginal crestal remodeling recorded at the implant neck ranged from 0.0 to 0.5 mm. Because this measurement was not prospectively standardized or assessed for repeatability, the range is not interpreted as a validated marginal-bone-loss or implant-success endpoint.

Fifty-two DENTIS OneQ Bone Level implants were placed and restored with fixed prostheses. Their dimensions are detailed in Table 4. All 52 implants remained in situ at the 1-year post-loading examination, giving descriptive implant survival of 52/52 (100%). All 20 patients retained every placed implant; the post hoc patient-level proportion was 20/20 (100%; exact 95% CI, 83.2–100%). No validated composite implant-success rate was calculated.

## 4. Discussion

### 4.1. Principal Findings

The principal clinical observation was that staged placement of two planned implants was achieved at all 26 treated sites and all 52 implants remained in situ through 1 year of loading. This provides preliminary evidence of feasibility for the described three-tooth extraction-site modification in a selected cohort. The radiographic means characterize the implant-bearing locations but do not establish individual dimensional stability. Morbidity was not negligible: half the patients developed a small-to-moderate hematoma, swelling was commonly pronounced, and pain lasted a mean of 4.7 days.

The operative clarification is important to interpreting this series: a single oral-side oval osteotomy outlines one segment containing the extraction sockets, and the entire segment is infractured superiorly. No separate upper cut or lateral window is made. Graft and gentamicin-containing collagen occupy the sockets and the space below the displaced segment, whereas the separate covering membrane is placed on the oral side. The study does not demonstrate preserved segment perfusion or an intact sinus membrane simply because this interface was not surgically dissected.

### 4.2. Relationship to Previously Published Techniques

The extraction-site procedures described by Jensen et al. [5] and Block [6] are particularly relevant predecessors because they use local alveolar bone in conjunction with molar removal. The present contribution is the clinical description and short-term follow-up of a contiguous segment spanning the second premolar through second molar, rather than introduction of sinus floor intrusion as a new principle.

Crespi et al. [7] compared two-step osteotome-mediated transalveolar sinus elevation with lateral palatal-wall displacement combined with alveolar ridge splitting in a three-year retrospective study of edentulous posterior maxillary sites. Their assessment included linear and volumetric comparison of CT examinations. In contrast, the present protocol mobilizes one contiguous three-tooth extraction-site segment and reports selected-point CBCT heights and implant survival through 1 year of loading. Differences in starting anatomy, movement of local bone, measurement dimensionality, treatment sequence, and follow-up prevent direct numerical comparison. The present single-arm series cannot establish an advantage over either approach or support conclusions about augmentation volume.

Classical segmental inlay and sandwich procedures also reposition local bone with interpositional grafting, but often treat an already edentulous ridge and use different segment displacement, grafts, or fixation [11,12,13,16,17,18]. Similarity of the operative concept does not demonstrate equivalent vascularity, membrane behavior, or regeneration. In particular, the present study contains no direct evidence that a specific pedicle maintained segment perfusion or that all radiopaque tissue represented vital bone.

### 4.3. Anatomical Selection and Radiographic Context

The contemporary sinus-augmentation literature supports anatomy-dependent planning rather than selection of one approach for every posterior maxillary defect [1,2,3,4]. The present series concerns adjacent teeth already requiring extraction and sufficient residual local bone to mobilize a segment. It does not establish an indication to extract otherwise maintainable teeth, or applicability to a severely resorbed edentulous ridge. The preoperative segment-height criterion should not be treated as a conventional residual subantral height threshold without a common anatomical definition.

Shetty et al. [21] developed a CBCT-based surgical-difficulty index for sinus augmentation, including sinus anatomy and pathology, and assessed agreement between readers. That pilot study concerns preoperative evaluation rather than the efficacy of a three-tooth segment procedure. Its relevance here is methodological: reproducible anatomical assessment and explicit sinus-pathology reporting are needed to define appropriate candidates and permit comparison between studies.

Current or previous sinus disease, including successfully treated sinusitis, and previous oroantral communication led to exclusion after otorhinolaryngological assessment. Eight of 53 assessed patients were excluded on these grounds. This conservative selection narrows generalizability and may have contributed to the favorable observed sinus-related clinical course. A further 25 of the 45 patients not excluded declined the operation because of its investigational nature and perceived greater invasiveness. Consecutive enrollment of the 20 consenting patients does not remove this preference-related selection. Their stated concerns are not evidence that the technique is objectively more invasive than conventional treatment; no comparative morbidity study was performed.

Anterior maxillary trials and posterior clinical reports provide useful background to segmental reconstruction, but differ in the defect treated, measurement definition, and duration of follow-up [13,14,15,16,17,18]. Their outcomes cannot serve as historical controls for the present selected cohort. Comparative studies would need to match both the starting anatomy and the intended prosthetic reconstruction.

### 4.4. Schneiderian Membrane and Sinus Safety

No clinically suspected membrane perforation was reported at 26 sites and no clinically suspected postoperative sinusitis or reoperation was reported in 20 patients. Intraoperative Valsalva testing and inspection for communication, followed by otorhinolaryngological review after primary healing, provide more information than an unspecified assessment of the postoperative course. Nevertheless, the membrane was not directly visualized during the procedure, and postoperative sinoscopy was not systematic. Endoscopically assessed experimental work and clinical syntheses underline the importance of ascertainment when reporting membrane injury [22,23]. The present observations cannot provide a verified membrane-perforation rate or exclude subclinical sinus disease.

A broad segment could distribute elevation forces differently from a focal instrument, but this remains a mechanical hypothesis. There were no pressure measurements, direct membrane observations, or comparison group. Grafting materials, local gentamicin-containing collagen, and the systemic prophylactic regimen were components of a treatment bundle; their individual effects on infection, healing, or implant retention cannot be separated in this series.

### 4.5. Interpretation of Radiographic and Implant Outcomes

The difference of 0.1 mm between the rounded follow-up means must not be interpreted as demonstrated stability of individual sites. Without paired measurements, dispersion, reproducibility assessment, and a reproducible alignment protocol, a small biological change cannot be separated from measurement variation or rounding. Selection of the highest intended implant-bearing point further limits inference to the whole reconstructed contour. Single-point height is neither augmented volume nor histological evidence of new vital bone. The 8 mm placement criterion and insertion of 10 or 12 mm implants also cannot independently verify mineralized-tissue height without quantitative implant-specific positioning data.

Implant survival was complete at the observed follow-up, but the 52 implants do not constitute 52 independent patients. The patient-level confidence interval of 83.2–100% makes the limited sample size explicit for the distinct endpoint of retaining all implants. It must not be interpreted as a cluster-adjusted implant-survival interval. Moreover, survival in situ does not establish peri-implant health, prosthetic success, or long-term durability.

Only DENTIS OneQ Bone Level implants were used. The investigators consider the underlying augmentation concept not inherently dependent on a particular implant brand; however, transferability to other systems was not tested. The institutional funder’s ownership relationship with a DENTIS distributor is disclosed under the Conflicts of Interest. Absence of financial or in-kind support from the distributor or manufacturer does not remove that potential competing interest.

### 4.6. Limitations

The uncontrolled design, small selected cohort, single center, and single surgeon restrict causal inference and generalizability. Bilateral sites and multiple implants introduce within-patient dependence. Screening flow is now reported, but exclusion of 8 candidates and refusal by 25 of the remaining 45 patients limit applicability beyond the 20 consecutive consenting participants. Refusal may reflect treatment preferences and perceived burden. The absence of registration limits verification of prospective outcome selection; neither internal funding nor the label “case series” resolves this issue [20]. The final calendar follow-up date is not available in the report dataset. Findings should not be extrapolated to smokers or patients with prior sinus disease, oroantral communication, diabetes, coagulation disorders, or other excluded conditions.

The principal measurement limitations are unavailable individual radiographic observations and dispersion, uncertain aggregation and contributing *n*, incomplete alignment and anatomical-reference documentation, undocumented blinding, single-point rather than volumetric assessment, lack of repeatability testing, and absence of histology. Acquisition parameters and the implant system are reported, but scan-level metadata were not independently verified during revision. Freehand individualized placement is documented qualitatively; quantitative implant-specific position and depth data are unavailable. The radiographic findings should therefore be treated as exploratory summaries, not as validation of anatomical gain or durable regional reconstruction.

Safety ascertainment did not include systematic visualization of the Schneiderian membrane or standardized diagnostic criteria for sinusitis. The timing and extent of early specialist investigation were incompletely documented, and clinical inspection cannot exclude silent injury. Morbidity assessment lacked validated pain-intensity, edema, and hematoma scales, and marginal remodeling was exploratory. Exact osteotomy depth, formal instability/rescue criteria, and the systemic antibiotic formulation and route were not available. Follow-up was limited to 1 year of loading. No authentic intraoperative photographic sequence or annotated longitudinal CBCT series is included; the conceptual figure cannot replace such clinical documentation. These limitations are not remedied by the favorable implant-retention count.

### 4.7. Future Research and Possible Applications

The next validation step should be a prospectively registered protocol with explicit anatomical eligibility, standardized clinical surveillance, and reproducible imaging. Complete recruitment and refusal reporting should be retained. Original site-level measurements should be preserved, repeatability quantified, and analyses account for bilateral sites and multiple implants. A comparative design should evaluate staged extraction and conventional augmentation as relevant alternatives. Direct assessment of membrane behavior, volumetric imaging, histology where justified, patient-reported outcomes, and longer loaded follow-up would address distinct questions not resolved by the present series.

Potential application is limited to selected patients requiring removal of three adjacent sinus-related posterior teeth. Combining extraction and augmentation while retaining a local bone segment may offer a practical reconstructive option, but comparative morbidity, safety, treatment burden, and effectiveness remain to be established.

## 5. Conclusions

In this prospective interventional case series of 20 consecutive eligible consenting patients, modified multi-tooth posterior maxillary alveolar segment infracture without a lateral window permitted staged placement of two implants at all 26 treated sites. All 52 implants remained in situ at 1 year after loading. These findings support preliminary feasibility of one-piece displacement of an extraction-site segment, not comparative superiority, confirmed membrane integrity, validated implant success, or individual radiographic stability. The eligibility restrictions and substantial pre-enrollment refusal limit generalizability. Further evaluation requires prospective registration, reproducible measurements, an appropriate comparison group, and longer follow-up.

## Figures and Tables

**Figure 1:**
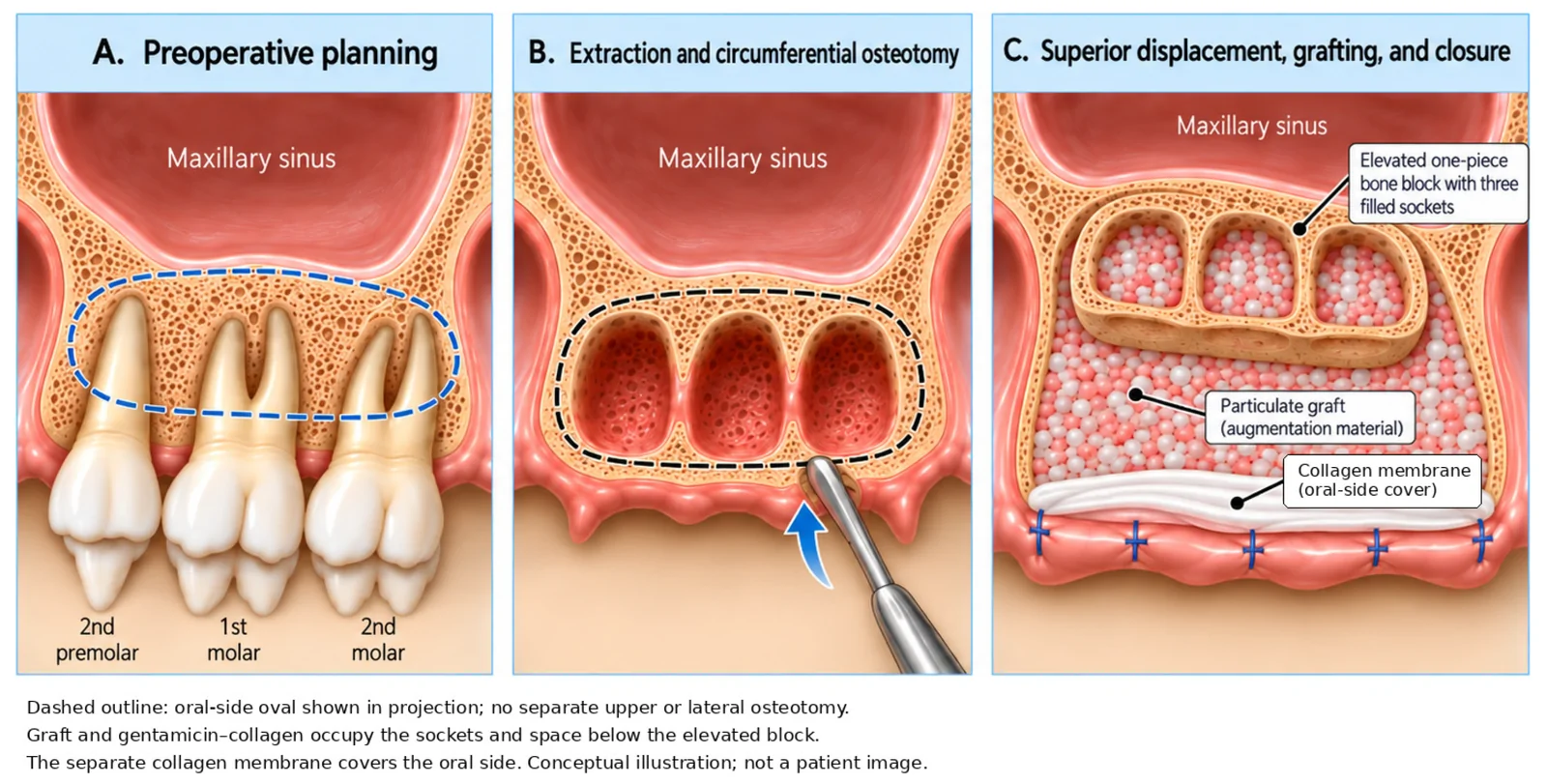
Conceptual illustration of one-piece posterior maxillary alveolar segment infracture and augmentation. (**A**) Planning of a contiguous segment spanning the second premolar, first molar, and second molar. (**B**) After extraction, a single oval circumferential osteotomy is made from the oral/crestal aspect around the three extraction sites. The dashed perimeter is a projected oral-side outline, not an additional superior or lateral-wall osteotomy. Controlled mallet taps through an osteotome mobilize the segment. The blue arrow in panel B indicates the direction of superior mobilization of the segment by the osteotome. (**C**) The entire block, including the socket walls, interradicular/interalveolar bone, and the three extraction-socket complexes, is displaced superiorly as one unit. The socket lumina remain open toward the oral augmentation compartment; the apparent rim in this oblique illustration does not represent a newly created inferior cortical plate. The sockets and the space beneath the elevated block are filled with particulate graft and gentamicin-containing collagen sponge. A separate resorbable collagen membrane covers the oral aspect, followed by soft-tissue closure. No membrane is placed between the block and the sinus. Implant placement after 6 months is not depicted. This author-directed, AI-assisted schematic is not to scale, is not a patient image, and does not document Schneiderian membrane integrity.

**Table 1:** Recruitment flow and baseline characteristics of the enrolled cohort.

Characteristic	Reported Value
Recruitment period	14 March–21 November 2023
Enrollment approach	Consecutive eligible consenting patients
Potential participants assessed	53
Excluded after otorhinolaryngological assessment	8
Declined the investigational operation before enrollment	25
Patients enrolled	20
Treated posterior maxillary sites	26
Men/women	12/8
Age, mean ± SD (range), years	62.3 ± 7.5 (47–79)
Unilateral/bilateral treatment	14/6 patients
Non-smokers	20/20 (100%)
Teeth extracted at each treated site	Second premolar, first molar, second molar
Minimum eligible preoperative segment height	7 mm

Screening and recruitment counts refer to patients: 53 assessed = 8 excluded + 25 declining participation + 20 enrolled. The 8 otorhinolaryngological exclusions are a combined count, not separate counts for each diagnosis. A treated site denotes one posterior maxillary side, not an independent patient or necessarily a separate operative session.

**Table 2:** Clinical outcomes and staged-placement feasibility, with reporting denominators.

Outcome	Unit	Result
Pain duration, mean ± SD	Patient	4.7 ± 2.3 days
Small-to-moderate hematoma	Patient	10/20 (50%)
Large hematoma	Patient	0/20
Clinically suspected postoperative sinusitis	Patient	0/20
Postoperative systemic antibiotics required	Patient	0/20
Reoperation	Patient	0/20
Loss to follow-up	Patient	0/20
Wound dehiscence	Treated site	0/26
Clinically suspected membrane perforation	Treated site	0/26
Two planned implants placed at 6 months	Treated site	26/26 (100%)

Site-level observations include bilateral treatment and are not statistically independent. No formal edema or hematoma severity scale was used. “Clinically suspected” reflects clinical ascertainment, not systematic endoscopic confirmation.

**Table 3:** Descriptive radiographic summaries; not paired or volumetric outcomes.

Variable	Reported Summary	Available Dispersion
Preoperative segment height	Mean 9.4 mm	Not available
Estimated segment displacement	Mean 7.2 mm	Not available
Implant-bearing mineralized-tissue height, 6 months after augmentation	Mean 12.9 mm	Not available
Implant-bearing mineralized-tissue height, 1 year after loading	Mean 12.8 mm	Not available
Exploratory marginal crestal remodeling	Range 0.0–0.5 mm	No SD reported

Height and displacement concern selected measurement locations within the 26-site cohort. The exact contributing *n* and aggregation across implant positions are unavailable; the means must not be treated as 26 independently verified observations each. Segment height, displacement, and follow-up mineralized-tissue height are not interchangeable measures of net bone gain. The reference baseline and measurement surfaces for exploratory marginal remodeling were not specified in the report dataset.

**Table 4:** DENTIS OneQ Bone Level implant dimensions; descriptive implant-level distribution.

Diameter × Length	Implants, *n*	Percentage
4.5 × 10 mm	4	7.7%
4.5 × 12 mm	13	25.0%
5.2 × 10 mm	23	44.2%
5.2 × 12 mm	12	23.1%
Total	52	100.0%

Implants were placed freehand with individualized geometry and are clustered within 26 treated sites and 20 patients. Dimensions do not establish individual augmentation height or complete intrabony accommodation.

## Data Availability

The aggregate results available for this report are presented in the manuscript. Individual paired radiographic measurements, their dispersion, and the detailed aggregation underlying the radiographic means are not available in the analyzed dataset. Requests concerning retained de-identified source records may be directed to the corresponding author; access is subject to record availability, appropriate permissions, and ethical and patient-privacy restrictions. Availability of a complete individual radiographic dataset is not claimed.

## Supplementary Materials

The following supporting information can be downloaded at https://www.mdpi.com/article/10.3390/jcm15197516/s1, Supplementary Material S1: Domain-based PROCESS 2023 reporting map. This map identifies reported items and residual reporting limitations and is not presented as certification of complete guideline compliance.
