# Supplementary material for: Clinical and Radiographic Outcomes of Modified Multi-Tooth Posterior Maxillary Alveolar Segment Infracture Without a Lateral Window for Staged Implant Placement: A Prospective Case Series
Source: J Clin Med. 2026 Sep 27;15(19):7516. doi: 10.3390/jcm15197516 (PMC13665869; doi:10.3390/jcm15197516)
Supplement: Supplementary file 1 [file jcm-15-07516-s001.zip › jcm-4494927-supplementary.pdf]

Domain-based PROCESS 2023 reporting map

Clinical and Radiographic Outcomes of Modified Multi-Tooth Posterior Maxillary Alveolar Segment Infracture Without a Lateral Window for Staged Implant Placement: A Prospective Case Series

This supplementary map locates the information reported in the accompanying manuscript and records residual reporting limitations. It is not the official item-by-item PROCESS checklist and does not certify complete guideline compliance. No study observations or analyses are added by this map.

1. Study identification and governance

Reporting domain	Location	Reported information and limitations
Title and design identification	Title; Abstract	The title identifies a prospective case series of modified multi-tooth posterior maxillary alveolar segment infracture without a lateral window and staged implant placement. The design is not presented as a controlled comparison.
Structured abstract	Abstract	Background/objectives, methods, results, and conclusions are reported. The abstract distinguishes patients, treated sites, and implants and limits conclusions to preliminary feasibility in selected patients.
Clinical rationale and evidence gap	Sections 1 and 4.2	The rationale is placed alongside extraction-site sinus intrusion, transcrestal augmentation, and segmental/sandwich osteotomy literature. The contribution is described as a modification involving one contiguous three-tooth segment, not a new biological principle.
Study design and setting	Section 2.1	Prospective, single-center, single-arm interventional case series at Asklepion-Lasercentrum, Prague, Czech Republic. Recruitment is reported as 14 March–21 November 2023.
Ethics and informed consent	Section 2.1; ethics and consent declarations	Asklepion Ethics Committee approval no. 2023/2, dated 15 February 2023, and written informed consent are reported. This map records the manuscript declaration; it is not a substitute for the original approval document.

2. Recruitment, intervention, and assessment

Reporting domain	Location	Reported information and limitations
Registration	Section 2.1; Section 4.6	No public trial registration was undertaken. The investigators' stated understanding at initiation is disclosed and is not presented as an exemption. The original outcome hierarchy and analysis plan cannot be independently verified against a prospectively registered protocol.
Participants and eligibility	Sections 2.2 and 3.1; Table 1	Fifty-three candidates were assessed: 8 were excluded on otorhinolaryngological grounds, 25 declined before enrollment, and 20 eligible consenting non-smokers were enrolled consecutively. Fourteen unilateral and six bilateral cases produced 26 treated sites. Eligibility and exclusions are described; refusal-related selection remains a limitation.
Intervention reproducibility	Sections 2.4–2.6; Figure 1	Extraction of three adjacent teeth, one oral-side oval osteotomy, superior mobilization of a single segment, grafting, oral-side membrane coverage, Wassmund flap advancement, aftercare, staged implants, and loading are described. Exact osteotomy depth, quantitative stability/rescue criteria, and the antibiotic formulation and route are unavailable.
Imaging and measurement	Section 2.3; Table 3	The reported device, acquisition settings, CAT vision version 3.0, timepoints, selected implant-bearing location, and comparison method are described. Scan-level metadata were not independently verified. Individual paired values, dispersion, exact aggregation, reproducible alignment details, and reliability analyses are unavailable.
Operator and evaluator roles	Sections 2.3–2.4; Author Contributions	Roman Šmucler performed the operations. Justina-Anna Šmuclerová (J.-A.Š.), who did not perform the operations, evaluated the radiographs and manually checked matching of measurement locations. Blinding to timepoint or clinical outcome was not documented. No independent reliability study is claimed.

3. Outcomes and analysis

Reporting domain	Location	Reported information and limitations
Outcomes and reporting units	Section 2.7; Tables 2–4	Staged implant-placement feasibility, mineralized-tissue height, implant retention, morbidity, and exploratory crestal remodeling are defined and reported with their units. Patient-, site-, and implant-level outcomes are distinguished. Retention in situ is not presented as a validated composite implant-success endpoint.
Sample size and statistical methods	Section 2.8	No a priori sample-size calculation was performed. Analyses are descriptive. Site/implant clustering is acknowledged; independence-based site/implant confidence intervals are not reported. The post hoc exact 95% interval for complete implant retention is patient-level. No unavailable individual observations, dispersion, or paired changes are imputed.
Follow-up and completeness	Sections 2.5–2.6 and 3.1	All 20 enrolled patients completed augmentation, implant placement, prosthetic rehabilitation, and examination 1 year after loading. No enrolled patient was lost. The final calendar follow-up date and exact timing/extent of early specialist review are unavailable. Pre-enrollment refusal is not loss to follow-up.
Adverse events and ascertainment	Sections 2.5, 3.2 and 4.4; Table 2	Pain lasted 4.7 ± 2.3 days; small-to-moderate hematoma occurred in 10/20 patients. No clinically suspected sinusitis or reoperation was reported in 20 patients, and no dehiscence or clinically suspected membrane perforation at 26 sites. Assessment was clinical, not systematic endoscopic verification; morbidity scales were not validated.
Implant and radiographic results	Section 3.3; Tables 3–4	All 26 sites received two implants and all 52 implants were retained at loaded follow-up. All 20 patients retained every implant (exact 95% CI, 83.2–100%). Radiographic means are descriptive; the 0.1-mm difference between rounded follow-up means is not a paired estimate or proof of individual dimensional stability.

4. Interpretation and transparency

Reporting domain	Location	Reported information and limitations
Discussion and comparison	Sections 4.1–4.5	Findings are compared qualitatively with related techniques. Anatomical selection, differences in measurement and follow-up, membrane ascertainment, and the distinction between radiopacity, vital bone, and implant retention are discussed. No comparative superiority or equivalence claim is made.
Limitations	Section 4.6	Limitations include the small selected uncontrolled cohort, single center/surgeon, within-patient clustering, non-registration, unavailable individual radiographic data, incomplete measurement documentation, non-systematic membrane assessment, limited morbidity metrics, absent histology, and short loaded follow-up. A synthetic diagram does not replace clinical documentation.
Future research and applications	Section 4.7; Conclusions	Prospective registration, reproducible imaging, full observation-level data, appropriate comparisons, direct membrane assessment, patient-reported outcomes, and longer follow-up are proposed. Potential application is restricted to selected patients already requiring extraction of three adjacent posterior teeth.
Patient perspective	Sections 2.2, 2.5, 3.2 and 4.6	Patient-reported pain duration and the stated reasons for declining participation are reported. A formal patient-perspective narrative or validated patient-reported outcome instrument is not reported. Their absence must not be replaced by inferred satisfaction or improved quality of life.
Data, funding, competing interests, and AI	Closing declarations	The available data are aggregate summaries; a complete individual radiographic dataset is not claimed. Internal funding by Asklepion-Lasercentrum and its ownership relationship with the DENTIS distributor ASKLEPION-Trade are disclosed, with no third-party support. AI assistance and the synthetic, non-clinical character of Figure 1 are disclosed.

Guideline referenced in the manuscript: Mathew, G.; Sohrabi, C.; Franchi, T.; Nicola, M.; Kerwan, A.; Agha, R.; PROCESS Group. Preferred Reporting of Case Series in Surgery (PROCESS) 2023 guidelines. Int. J. Surg. 2023, 109, 3760–3769. doi:10.1097/JS9.0000000000000940.
